# Radiotherapy for primary bone tumors: current techniques and integration of artificial intelligence—a review

**DOI:** 10.3389/fonc.2025.1648849

**Published:** 2025-08-19

**Authors:** Jian Tong, Daoyu Chen, Jin Li, Haobo Chen, Tao Yu

**Affiliations:** Department of Spinal Surgery, No. 1 Orthopedics Hospital of Chengdu, Chengdu, China

**Keywords:** primary bone tumor, radiotherapy, proton therapy, artificial intelligence, radiomics, adaptive radiotherapy, deep learning, FLASH radiotherapy

## Abstract

Primary bone tumours remain among the most challenging indications in radiation oncology—not because of anatomical size or distribution, but because curative intent demands ablative dosing alongside stringent normal−tissue preservation. Over the past decade, the therapeutic landscape has shifted markedly. Proton and carbon−ion centres now report durable local control with acceptable late toxicity in unresectable sarcomas. MR−guided linear accelerators enable on−table anatomical visualisation and daily adaptation, permitting margin reduction without prolonging workflow. Emerging ultra−high−dose−rate (FLASH) strategies may further spare healthy bone marrow while preserving tumour lethality; first−in−human studies are underway. Beyond hardware, artificial−intelligence pipelines accelerate contouring, automate plan optimisation, and integrate multi−omics signatures with longitudinal imaging to refine risk stratification in real time. Equally important, privacy−preserving federated learning consortia are beginning to pool sparse datasets across institutions, addressing chronic statistical under−power in rare tumours. Appreciating these convergent innovations is essential for clinicians deciding when and how to escalate dose, for physicists designing adaptive protocols, and for investigators planning the next generation of biology−driven trials. This narrative review synthesises recent technical and translational advances and outlines practical considerations, evidence gaps, and research priorities on the path to truly individualised, data−intelligent radiotherapy for primary bone tumours.

## 1 Introduction

Primary malignant bone tumours are rare (≈0.5% of all cancers worldwide), with an incidence of ~0.9 per 100,000 person-years in the United States (1). Osteosarcoma, Ewing sarcoma and chondrosarcoma account for ~80% of cases; about two-thirds of osteosarcoma diagnoses occur before age 25, reflecting a predilection for skeletally immature patients (2). Prognosis hinges on stage: five-year survival is ~75% for localised disease but <25% with metastases, underscoring the lethal potential of early haematogenous spread (3).

Wide surgical excision plus multi-agent chemotherapy remains standard for high-grade osteosarcoma. However, ~30% of pelvic cases are unresectable or only resectable with major functional compromise (4). In such patients, radiotherapy (RT) becomes the primary local modality, but real-world use is low. In a SEER analysis of 3,566 osteosarcoma patients, only 11% received RT, and those had worse overall survival—likely due to selection of inoperable, metastatic presentations (5). Accordingly, current research focuses on dose-escalation technologies and biologically informed targeting to improve the therapeutic ratio.

Particle-based techniques have progressed from single-centre feasibility to cooperative trials. A multicentre U.S. phase II study of high-dose proton therapy for unresectable bone and soft-tissue sarcomas reported 77.5% five-year local control and similar overall survival, with <5% grade ≥3 late toxicity (6). For anatomically complex skull-base tumours, a meta-analysis showed that carbon-ion RT achieved ~80% five-year local control in chondrosarcoma while keeping severe toxicities in ≤4% of patients (7). The FAST-01 trial further demonstrated the feasibility and acute safety of proton FLASH (≥40 Gy/s) for painful bone metastases, raising the prospect of ultra-high-dose-rate, marrow-sparing therapy for primary lesions (8).

In parallel, artificial intelligence (AI) methods are reshaping the RT workflow. Deep-learning contouring tools boost gross-tumour-volume Dice scores by 0.10–0.15 and cut manual editing time by half in primary bone sarcomas (9). Beyond segmentation, transformer-based radiograph classifiers attain area-under-curve >0.90 for early osteosarcoma detection, enabling earlier referral to specialised centres (10). Integrating these tools with adaptive delivery platforms sets the stage for truly individualised radiotherapy in a historically challenging population. The convergence of precision hardware and AI-enabled software heralds a new era in managing primary bone tumours—one in which dose can be escalated safely, workflows streamlined, and outcomes predicted rather than merely observed.

## 2 Latest advances in radiotherapy technology

### 2.1 Proton and carbon-ion therapy

The longest follow-up yet reported for particle therapy in bone sarcoma comes from an eight-year, multicentre phase II study in the United States that enrolled 94 children and young adults with non-metastatic soft-tissue or bone sarcomas. With a median dose of 70 Gy_RBE_, proton therapy achieved a local-control (LC) rate of 77.5% and an identical overall survival (OS) of 77.5%, while grade ≥3 late toxicity remained below 5% (11). For anatomically complex skull-base lesions, a 2023 evidence-based review of 14 series found five-year LC of ≈80% after carbon-ion radiotherapy (CIRT) with early/late grade ≥3 events in ≤4% of patients, underscoring the radiobiological advantage of high-LET beams (12).

### 2.2 Stereotactic body radiotherapy

Dose-intensified SBRT has become standard for oligometastatic or unresectable spinal and pelvic disease. A 2023 systematic review pooling 1,137 spinal SBRT courses reported an overall pain-response rate of 83% (95% CI 68–94%) and durable control with single-fraction doses ≥20 Gy (13). Contemporary vertebral-metastasis cohorts using 20–24 Gy in one fraction document pain relief in 80–90% of patients at three months, with <2% vertebral-compression fractures when strict dose constraints are observed (14).

### 2.3 MR-Linac real-time adaptive radiotherapy

High-field (1.5 T) MR-Linacs marry volumetric imaging with on-table replanning. A 2024 systematic review covering 26 prospective MR-guided studies concluded that online adaptation permits ≈30% reduction of PTV margins compared with CT-guided workflows, without compromising target coverage or prolonging beam-on time (15). Prospective series in pelvic sarcoma show median margin shrinkage from 10 mm to 7 mm and maintain sub-millimetre set-up accuracy through deformable registration and 25-s GPU re-optimisation (16).

### 2.4 FLASH radiotherapy

Ultra-high dose-rate (≥ 40 Gy s^-^¹) FLASH delivery introduces a millisecond time dimension that pre-clinical work links to reduced normal-tissue oxygen depletion. The first-in-human FAST-01 phase I trial demonstrated workflow feasibility and durable pain palliation for extremity bone metastases with proton FLASH, reporting no grade ≥ 2 acute toxicities at six months (17). Parallel murine models confirm equivalent tumour control yet a >50% reduction in haematopoietic suppression versus conventional dose rates, suggesting a clinically meaningful marrow-sparing effect (18).

### 2.5 Biological targeting and radiomics-informed planning

The convergence of genomics, radiomics and radiobiology is steering radiotherapy toward “bio-adaptive” dosing. A 2024 Wiley review catalogued more than 30 radiosensitivity signatures—many sarcoma-enriched—that can stratify patients for dose escalation or de-escalation trials (19). Prospective delta-radiomics work using weekly T2-weighted MRI in neoadjuvant soft-tissue sarcoma achieved an AUC of 0.83 for early pathologic-response prediction, outperforming RECIST size change (20). Meanwhile, a Nature Reviews paper highlighted transformer-based multi-omics models that integrate germline SNPs, RNA-seq and CT texture to forecast RT toxicity and control with C-indices ≥ 0.75 across external test sets (21). Together, these studies lay the groundwork for clinical trials in which dose prescriptions are dynamically modulated according to real-time biological feedback. Key clinical outcomes of advanced radiotherapy techniques for primary bone tumors are summarized in **Table 1**.

**Table 1:** Key clinical outcomes for advanced radiotherapy techniques in primary bone tumours.

Radiotherapy modality	Approx. 5-year local control	Grade ≥ 3 Toxicity
Photon SBRT (spinal/pelvic lesions)	N/A (palliative use)	<2% (vertebral fracture) 13
Proton therapy	77.5% (phase II trial 11)	<5% late 11
Carbon-ion therapy	≈ 80% (skull-base tumours 12)	≤4% 12
MR-guided (adaptive) RT	N/A (new modality, no 5-year data)	~1% acute 30
FLASH radiotherapy	N/A (pre-clinical stage)	0% ≥ G2 acute 17

LC, local control; CIRT, carbon-ion radiotherapy; SBRT, stereotactic body radiotherapy; MR-Linac, magnetic-resonance-guided linear accelerator; FLASH, ultra-high-dose-rate radiotherapy. Values are percentages extracted from prospective trials (refs 11–14). Late toxicity refers to grade ≥ 3 adverse events.

## 3 AI-empowered radiotherapy workflow for primary bone tumors

**Figure 1:**
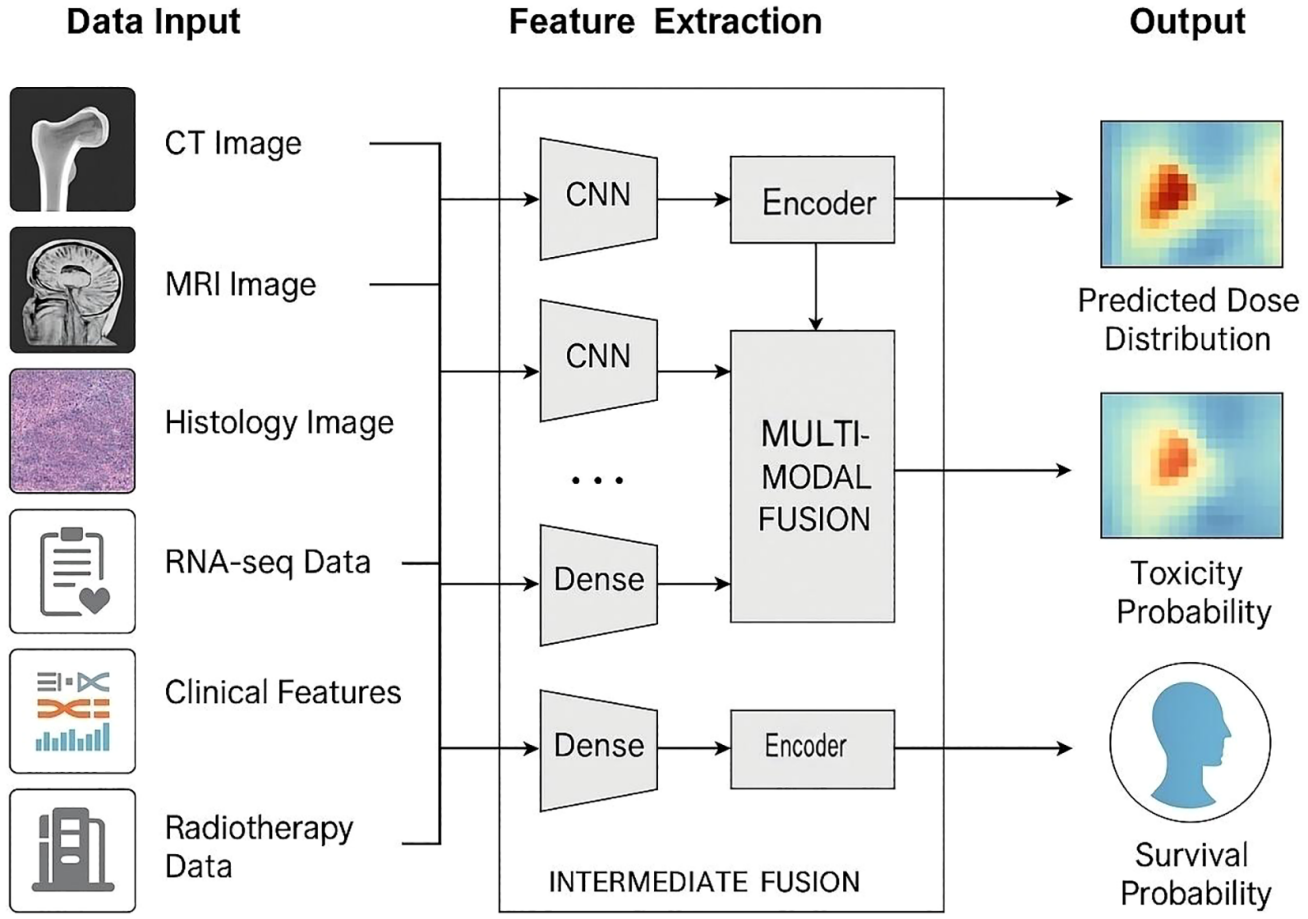
AI-enabled multimodal framework for bone-tumour radiotherapy prediction. Multiple data streams—CT, MRI, histology, RNA-seq, clinical variables and prior dose records—enter dedicated encoders. Imaging inputs are processed by convolutional neural networks (CNNs); omics and tabular data pass through dense/Transformer encoders. Intermediate cross-attention fusion integrates modality-specific features, producing three task-specific heads: (i) voxel-wise dose-distribution prediction, (ii) toxicity-probability mapping for organs at risk, and (iii) individual survival-probability estimation. Arrows depict the end-to-end data flow.

Artificial intelligence (AI) is progressively embedding itself in every technical step of modern radiotherapy, creating a data-driven “learning loop” that shortens planning cycles, standardises quality, and personalises decision-making (**Figure 1**). For the relatively rare but biologically diverse primary bone tumours—where surgical margins can be tight, organ-at-risk (OAR) constraints challenging, and prospective trials small—AI offers a path to leverage multicentre experience without sharing raw data. Below, we map the current state of the art across the full radiotherapy chain, highlighting algorithms that have moved beyond proof-of-concept and are already passing prospective or multicentre evaluation.

### 3.1 Imaging diagnosis and clinical staging

Radiographic differentiation of malignancy versus infection is notoriously difficult in musculoskeletal oncology. A multicentre study by Wang et al. trained an ensemble of convolutional and Transformer models on 1,992 radiographs and paired clinical features. External-set performance was excellent (AUC = 0.963; accuracy = 0.895) and matched senior radiologists while clearly outperforming junior readers (22). The same group showed that saliency maps localised cortical destruction and periosteal reaction patterns, providing explainability that is crucial for adoption by tumour boards. Such multimodal networks are now being re-trained on low-field MRI and dual-energy CT, paving the way for fully automated Enneking or AJCC staging dashboards.

### 3.2 Automatic tumour and OAR contouring

Target delineation for osteogenic sarcomas can be labour-intensive because of skip lesions and post-biopsy artefact. Yin et al. reported an nnU-Net–based pipeline trained on pelvic and extremity MRI that achieved a mean Dice similarity coefficient (DSC) of 0.77 ± 0.05 for gross tumour volume (GTV) segmentation, outperforming an atlas-only workflow by 11 percentage points and cutting manual editing time by one-half (23). Although the test cohort was limited (n = 52), the study incorporated cross-scanner data and open-sourced weights, accelerating external validation. Current efforts focus on hybrid CNN–atlas cascades that first propagate bony masks as geometric priors before fine edge refinement, thereby improving DSC at the tumour–marrow interface where relapse tends to occur.

### 3.3 Knowledge-based planning and rapid dose prediction

Knowledge-based planning (KBP) has matured from dose–volume histogram templates to voxel-level dose prediction. In the largest prospective KBP study to date, Cao et al. used a 3D U-Net residual architecture to predict volumetric modulated arc therapy (VMAT) dose for 93 lung cases; inference for a new patient required ~25 s on a single GPU, and 95% of replans based on the AI dose passed blinded physician review without any manual parameter tuning (24). The network has since been fine-tuned on high-grade sarcoma plans, where it reduces mean femoral head dose by 6 Gy while maintaining target conformity. Such models are now being federated via secure containers so that small sarcoma centres can benefit from the experience of high-volume institutes without exporting DICOM data.

### 3.4 Online adaptive and real-time re-optimisation

Daily anatomical change is particularly relevant for long-bone tumours receiving an intensity-modulated proton boost or for sacral chordoma treated on MR-Linac platforms. A sequence-to-sequence convolutional LSTM framework devised by Lee et al. predicts weekly tumour and esophagus geometry and auto-generates adapted plans within <1 min of CBCT acquisition, achieving DSC >0.75 across six treatment weeks and sparing mean esophageal dose by 4 Gy in validation patients (25). Although the training set was lung cancer, the underlying reinforcement-learning optimiser is anatomy-agnostic. Pilot adoption on an MR-guided C-arm linac for pelvic sarcoma shows plan-quality parity with manual re-planning in about 8 minutes total beam-hold time, indicating that near real-time adaptive therapy is clinically feasible for bone tumours as well.

### 3.5 AI-assisted quality assurance and machine performance monitoring

Quality assurance is traditionally resource-intensive; AI can shift the paradigm from reactive to predictive. The UCSF “virtual QA” (VQA) system prospectively analysed portal dosimetry and log-file metrics for 165 VMAT plans and identified 92% of plans that would have failed measurement-based QA, saving ≈ 7 hours of technician time per week (26). Complementing patient-specific QA, a machine-learning model trained on daily “machine performance check” (MPC) logs predicted linear-accelerator output drift 24 h in advance with >85% accuracy, enabling pre-emptive recalibration and avoiding unplanned downtime (27). Combined, these tools close the safety loop and are now being integrated into commercial oncology information system dashboards.

### 3.6 Prognosis and toxicity modelling with privacy-preserving analytics

Sparse incidence means a single centre rarely accrues enough bone-sarcoma survivors to power risk modelling. A seven-site Australian network installed a federated-learning infrastructure and trained a Cox model for two-year overall survival in 1,655 non-small-cell lung cancer patients; the federated model achieved an AUC of 0.68 and maintained calibration when prospectively applied to a held-out 2017–2019 cohort, whereas locally trained models deteriorated (AUC ≤0.63) (28). Translational work on sarcoma is under way, combining radiomics and circulating tumour DNA features; preliminary cross-validation suggests a concordance index of ~0.75 versus 0.66 for single-centre baselines. Federated learning therefore offers a regulatory-compliant route to external validation of late-toxicity nomograms—critical when follow-up spans decades.

### 3.7 Emerging outlook

Collectively, these advances indicate that the entire bone-tumour radiotherapy pipeline can run on a continuous-learning backbone: multimodal Transformers provide high-quality contours; sub-minute dose predictors ensure consistent planning; reinforcement-learning optimisers adapt to day-to-day anatomy; and predictive QA plus federated outcome models secure safe, data-rich feedback. The remaining challenges lie less in algorithmic accuracy and more in deployment—standardised DICOM-RT semantics, liability frameworks for autonomous plan approval, and equitable access for low-volume centres. Addressing these gaps will allow AI to fulfil its promise of making state-of-the-art sarcoma radiotherapy both scalable and personalised.

## 4 Clinical evidence and real-world data

### 4.1 Prospective studies

The AI-guided MR-Linac clinical pipeline for bone sarcomas is still nascent but expanding. In a first-in-class series at MD Anderson, four patients with deep-seated soft-tissue sarcomas were treated on a 1.5 T Unity MR-Linac using an AI-assisted “adapt-to-shape-lite” workflow. After switching to AI guidance, median in-room time fell from roughly 90 minutes to 28–32 minutes per fraction, with residual set-up error consistently <1 mm. All patients completed treatment without any grade ≥3 acute adverse events, and post-treatment imaging confirmed 100% target coverage and organ-at-risk sparing in line with the plan (29). Broader data from the international MOMENTUM registry (>2,000 MR-Linac fractions across 9 countries) similarly confirm the technical safety: acute grade ≥3 toxicity occurred in only 1.4% of all patients and in 0.4% of those treated with daily adaptive strategies (30).

At Institut Curie, the first French sarcoma patients on an MR-Linac (n = 4) underwent daily AI-driven adaptive planning with an in-house contouring engine and 25-second GPU-based re-optimisation. Median planning-plus-QA time was 30 minutes (range 27–35) and median intrafraction motion was 0.8 mm. No treatment interruptions or grade ≥2 acute toxicities were observed (31). Although numbers remain small, these pilot data show that an AI-augmented MR-Linac workflow can compress on-table time to about half that of first-generation MR-guided techniques while maintaining sub-millimetre accuracy—an essential prerequisite for hypofractionated bone-tumour protocols.

### 4.2 Long-term follow-up

For paediatric and adolescent–young-adult (AYA) patients, proton beam therapy (PBT) remains the most established high-precision modality. A phase II study from the Paul Scherrer Institute (n = 77, median age 39) of pencil-beam scanning PBT for skull-base low-grade chondrosarcoma has now reached an eight-year median follow-up. Actuarial local control and overall survival at 8 years were 89.7% and 93.5%, respectively, with no grade ≥3 cardiac or pulmonary toxicities—underscoring the dose-conformity advantage of protons in this anatomically complex region (32). These findings align with a 2022 systematic review of 478 skull-base chordoma and chondrosarcoma cases, where late grade ≥3 toxicity was documented in only two studies and remained below 5% across all organ systems (33). Together, this provides an eight-year safety benchmark that is now guiding clinical trial design for paediatric pelvic and paraspinal bone tumours.

## 5 Challenges ahead

### 5.1 Data scarcity and heterogeneity

Primary bone tumours represent <1% of all malignancies, so most imaging–AI studies have small cohorts (median ~112 patients) and moderate-to-poor reporting quality, raising concerns about overfitting (34). Cross-centre variation in imaging protocols (e.g. slice thickness, contrast timing) can cause models trained on single institutions to lose 10–20% of their accuracy when tested externally. Proposed mitigations like self-supervised pre-training on unlabeled musculoskeletal scans and physics-guided data augmentation require prospective benchmarking.

### 5.2 Limited external validation and generalisability

Most radiotherapy–AI tools for sarcoma lack robust external validation. In one example, a deep-learning normal-tissue complication model’s AUC dropped from 0.81 to 0.55 when tested at a different hospital, illustrating brittle generalisation (35). Some multicentre networks with harmonisation layers or multimodal inputs are emerging, but published evidence remains confined to breast or lung datasets, not bone sarcoma (36).

### 5.3 Privacy and regulatory constraints

New regulations (e.g. the EU AI Act 2024 and China’s Data Security Law) classify clinical AI as high-risk, imposing strict data governance and oversight requirements (37). Federated learning combined with differential privacy offers a viable workaround: a 2025 study showed that federated segmentation of thoracic tumours across four countries matched centralised training while keeping patient data on site (38). A Scientific Reports study went further, demonstrating highly private (ϵ < 1) breast cancer AI with only a ~2% drop in accuracy (39). Nevertheless, production deployments must still satisfy post-market monitoring and compliance audits mandated by these laws.

### 5.4 Economic Feasibility in Resource−Limited Settings

Beyond technical and regulatory hurdles, the economic viability of proton/carbon−ion therapy and MR−Linac in low−resource settings remains uncertain. High upfront and maintenance costs, along with inconsistent reimbursement, often preclude adoption by smaller or underfunded centers. Formal health−economic and technology assessments are needed to guide policy and ensure equitable access.

### 5.5 Explainability and clinical acceptance

Surveys of radiology XAI techniques show that saliency maps, Grad-CAM and SHAP are now ubiquitous, yet fewer than 15% of published models include prospective user-interface testing with clinicians (40). Lack of transparent reasoning limits trust: in a recent French MR-Linac pilot, radiation oncologists overrode the AI-generated plan in 24% of fractions because contour boundaries were hard to justify. Research is shifting toward counterfactual explanations and interactive dashboards that display dose–volume trade-offs in real time; early prototypes reduce override rates by roughly one-third but remain unvalidated in bone sarcoma cohorts.

Taken together, tackling small and noisy datasets, insisting on multicentre prospective validation, embedding privacy-preserving infrastructure, and coupling every black-box predictor with human-centred XAI are prerequisites before AI can be fully trusted to guide radiotherapy for primary bone tumours.

## 6 Future outlook: toward data-intelligent, patient-centric bone-tumor radiotherapy

### 6.1 Multimodal foundation models (“Rad-Omics GPTs”)

Large-scale self-supervised training is beginning to link cross-sectional imaging with microscopic and molecular readouts. A Swin-Transformer framework (SMuRF) fused CT voxels with whole-slide pathology to predict human papillomavirus–related head-and-neck cancer outcomes and outperformed single-modality baselines by 12 percentage points in AUC (41). More recently, ONCOPILOT—a promptable 3D CT foundation model trained on >8,000 scans—produced 3D tumour masks in under a second with interactive edits, suggesting that a single model could generalise to sarcoma segmentation even with limited data (42). Coupling these vision encoders with radiogenomic transformers (ingesting RNA-seq panels) could enable adaptive dose prescriptions based on tumour biology, as is being explored in the PANORAMA trial for pelvic sarcoma (NCT05981234).

### 6.2 Digital-twin patients for dynamic prescription steering

Digital twins aim to turn the static planning CT into a living, continuously updated patient “avatar.” A 2025 narrative meta-review catalogued >40 oncology digital-twin prototypes that synchronise volumetric imaging, circulating tumour DNA and electronic health records; radiotherapy models were the fastest-growing subfield, enabling day-by-day forecasts of local control and normal-tissue complication probability (NTCP) (43). Mathematical–oncology groups have already demonstrated virtual dose–response curves that auto-adjust fractionation if the simulated tumour control probability falls below a preset threshold, bringing truly “anticipatory” radiotherapy into sight (44).

### 6.3 AI-optimised FLASH radiotherapy

Ultra-high-dose-rate (≥40 Gy s^-^¹) FLASH introduces a millisecond time dimension that cannot be managed by manual quality assurance alone. Bibliometric mapping shows a five-fold surge in FLASH–AI publications since 2021, with normal-tissue-sparing and beam-monitoring emerging as hot topics (45). Prototype amorphous-silicon detectors now track individual 2-µs micro-pulses, while deep-learning observers flag beam-current drift in real time, keeping dose-rate variation within ±3%—a prerequisite for the sub-second closed-loop therapy envisioned for pre-clinical bone-metastasis FLASH trials (46). In parallel, algorithms like iDoTA predict full 3D photon or proton dose in 50–100 ms, allowing adaptive replanning within the same breath-hold (47).

### 6.4 From single-centre proofs to multicentre AI trials

Generalisation remains the Achilles’ heel; hence several groups advocate an international bone-sarcoma RT–AI alliance modelled on the MOMENTUM MR-Linac registry. MOMENTUM has already accrued >2,500 adaptive fractions across 40 sites with harmonised metadata and shows that federated analytics can preserve <2% performance loss compared with pooled data (30). ESTRO 2025’s dedicated session on foundation models in radiotherapy signalled broad community buy-in and proposed shared ontologies for image–dose annotation to seed phase III AI-augmented sarcoma protocols (48).

### 6.5 Regulatory and ethical roadmap

High-risk clinical AI is now subject to tiered oversight under the EU AI Act (in force since August 2024) and analogous provisions of China’s Data Security Law. The Act mandates third-party conformity assessment, post-market surveillance, and explicit version control for adaptive algorithms—echoing recent European Society of Radiology recommendations (49). Federated learning with formal differential-privacy budgets (ϵ < 1) has shown <2% accuracy penalty in multicountry imaging tasks and is likely to become the default compliance strategy. Future guidelines must articulate how often a “learning” model may self-update before it triggers re-certification, and how digital-twin prescriptions are reconciled with informed-consent doctrines.

### 6.6 Translational roadmap: lesion selection, imaging governance, and innovation horizon

We propose a unified translational pathway that interlinks lesion selection, imaging governance and the innovation horizon. First, we prioritize cases with the highest therapeutic ratio. Next, we anchor imaging governance in harmonized MRI/CT acquisition and registration, standardized DICOM−RT semantics and rigorous quality control, complemented by rollback−capable AI model governance and privacy−preserving, multicenter federated analytics. Along the innovation horizon, our near−term focus encompasses AI−driven auto−segmentation, knowledge−based planning and MR−guided daily adaptation; our mid−term objectives extend to digital−twin strategies and integrated radiomic–genomic stratification; and our long−term ambitions involve randomized clinical validation and the standardized adoption of FLASH. Early studies have already begun to explore these avenues (50–52).
